# Assessment of an electronic voting system within the tutorial setting: A randomised controlled trial [ISRCTN54535861]

**DOI:** 10.1186/1472-6920-5-24

**Published:** 2005-07-07

**Authors:** Edward J Palmer, Peter G Devitt, Neville J De Young, David Morris

**Affiliations:** 1 Centre for Learning and Professional Development, University of Adelaide, South Australia; 2 Department of Surgery, University of Adelaide, South Australia; 3 Department of Obstetrics and Gynaecology, University of Adelaide, South Australia

## Abstract

**Background:**

Electronic voting systems have been used in various educational settings with little measurement of the educational impact on students. The goal of this study was to measure the effects of the inclusion of an electronic voting system within a small group tutorial.

**Method:**

A prospective randomised controlled trial was run at the Royal Adelaide Hospital, a teaching hospital in Adelaide, Australia. 102 students in their first clinical year of medical school participated in the study where an electronic voting system was introduced as a teaching aid into a standard tutorial. Long-term retention of knowledge and understanding of the topics discussed in the tutorials was measured and student response to the introduction of the electronic voting system was assessed.

**Results:**

Students using the electronic voting system had improved long-term retention of understanding of material taught in the tutorial. Students had a positive response to the use of this teaching aid.

**Conclusion:**

Electronic voting systems can provide a stimulating learning environment for students and in a small group tutorial may improve educational outcomes.

## Background

In the modern teaching environment, the small group tutorial is favoured over lectures. A well run tutorial is stimulating and involves frequent participation from all members within the class in a non-threatening environment. Perhaps the main advantage of a small class size is the ability to incorporate a number of active learning strategies. The inclusion of discussion methods can lead to improved student performance [1,2], particularly when considering retention of knowledge, critical thinking and problem solving [3]. Unfortunately, it is a common observation that tutors tend to use the same teaching methods whether they are delivering lectures or running tutorials. This can result in dry and lifeless tutorials, devoid of any student participation and wasting the great potential for active learning implicit in the format.

Several factors influence the dynamics of a tutorial. The style and attitude of the tutor is critical and those who seek to engage their group in a non-threatening manner are more likely to be rewarded by increased group participation. The larger the group, the more difficult will be the engagement. This is partly because of the difficulty in involving many people in one discussion, but also the reluctance of individuals to potentially be embarrassed in front of their peers. Students need to feel that they can trust the tutor and their fellow students before they will contribute [4]. Another consideration is the cultural background and gender of the students, which has a bearing on the nature of participation in groups [3,5]. The mix of tutor and student personality as well as culture may have a considerable impact on the dynamics of any tutorial in a multicultural environment and even with the best endeavours of the tutor, getting contributions from all members of the group may be exceedingly difficult.

Many strategies exist to try and improve individual participation. We have undertaken a study to assess the place of an electronic voting system in the dynamics of a tutorial. We have set out to observe the effectiveness of such a system during a tutorial, to record the opinions of the students on the value of the system and to measure what was learnt and understood during the tutorial.

At its simplest, an electronic voting system (EVS) consists of a master control unit and a series of hand held devices, which communicate with the controller. Audience members, students in this case, push one or more of these buttons in response to a question posed by the tutor. A computer allows the tutor to examine and display the information gained from the hand held units. In this way, the tutor and students receive instant feedback about the student responses to the question.

The hypotheses to be tested are that the introduction of an electronic voting system (EVS) into a class environment enhances enjoyment of the learning experience and improves understanding and retention of knowledge of the material presented using the system in both the short and long-term.

## Methods

102 students enrolled in the study. All of the students were in the age range 21–23 and had completed three years of a six-year undergraduate medicine programme. This was their first year of clinical experience. All students in the study came from one academic year and represented 93% of the students in that year. The students were randomly allocated into one of six groups and stratified for gender and academic ability (i.e. previous academic performance in the medical course). The randomisation was carried out by the secretariat in the medical school, who had no connection with the study. In order to verify that this randomisation had been carried out effectively, a one-way ANOVA was used to look for differences between groups in the areas of gender and academic ability. Academic ability was measured using the previous years exam results.

The study was carried out at six different times during the year as groups of 13–21 students rotated through the surgical component of their year. It consisted of a pre-test evaluation of the students' prior knowledge and understanding of two topics followed by a tutorial on each topic. At the end of each tutorial there was a post-test evaluation and a questionnaire. Six weeks after the tutorials, the post-test evaluation was repeated. The tests for each topic had two components; a knowledge test of 11 multiple-choice questions and two multi-stage problem-solving questions designed to measure understanding. The tests did not contribute in any way to the overall student assessment for their degree.

The two tutorials were on the topics of acute abdominal pain and gastrointestinal haemorrhage. A series of clinical simulations were constructed and used as the basis for each tutorial. The simulations were computer-based and delivered using a data projector, and a computer program, *Medici*, which has been described previously [6,7]. Each tutorial was approximately 45 minutes long and covered three different clinical scenarios. The use of the computer material was similar for the two tutorial subjects, with or without the use of EVS. Due to technical issues, the EVS was always applied in the second half of the teaching session.

Students were randomised into two groups. The randomisation was done independent of the study and stratified students for gender, ethnic origins and academic ability. One group had the topic of acute abdominal pain discussed in tutorial format alone followed by the second topic of gastrointestinal haemorrhage, which was a tutorial supplemented with an electronic voting system. In the other group, the topic of gastrointestinal haemorrhage was discussed in tutorial format with the acute abdominal pain topic tutorial following and supplemented with an electronic voting system.

The electronic voting system was developed by one of the authors (DM) and has been used as part of a telemedicine system to aid postgraduate training in obstetrics [8]. Each member of the tutorial group was provided with a hand-held keypad and asked to select the number corresponding to their choice at various stages of the tutorial. Each keypad was connected by wires to a central control box, and the accumulated data fed into a computer. A software program collated and displayed in histogram form, the accumulated responses for each choice provided by the students.

The case simulations in the Medici program provide a clinical scenario on which discussion develops. Several diagnostic or management options are displayed and the members of the tutorial group invited to use their keypads to make a selection. The combined group selections are then displayed with the EVS software. Further discussion promoted by the tutor then takes place, based on the histograms.

The behaviour and interactions between students was formally monitored by one of the authors (EP) during the tutorial sessions. A second independent observer was used to check the results of the primary observer. The results of these observations were checked at the end of each session. Where differences were noted, the results of the observer closest to the interaction were used. The number of interactions between lecturer and students and the level of each interaction was recorded and classified according to the schema shown in Table 1. A Level 2 interaction would be considered a baseline for a meaningful student-lecturer dialogue. During 5-minute 'snapshots, other student activity (writing notes, talking between themselves) was recorded. Also noted were the behaviour of the class as a whole and the attitudes of students and lecturer. At the end of each tutorial, the students' attitudes towards the use of the EVS were measured.

**Table 1:** Schema for assessing levels of interaction

Level 1	Simple Interaction eg a yes/no answer to a question from a student or tutor
Level 2	A short answer (<1 min) to a question from either student or tutor
Level 3	An extended interaction of at least 1 minute creating further discussion

The instrument used to measure the student's opinion of the introduction of the EVS was a six-item questionnaire. Each item was scored on a five-point scale (strongly disagree, disagree, no opinion, agree and strongly agree). It measured the reaction of the student to the use of the EVS (was it worthwhile, did it aid participation, was it a distraction, did it aid understanding). The scale was computed by summing the ratings for the six items. The internal consistency of the scale was determined using the Cronbach's Alpha statistical test (SPSS)

Comparisons of the results of observing student dynamics were carried out using the Mann-Whitney test. The results of the pre, post and long-term tests of knowledge and understanding were compared using a mixed model repeated measures analysis. Pairwise comparisons of the impact of EVS at each combination of time (pre-test, post test and 6 week test) and order (acute abdomen (no EVS), gastrointestinal haemorrhage (EVS) or gastrointestinal haemorrhage (no EVS), acute abdomen (EVS)) were made using Student's t-test. The impact of gender of students and their educational status (international student or Australian student) was assessed.

## Results

In the six groups, there were a total of 102 students. 99 students completed the questionnaire and 86 completed all three elements of the pre- and post-testing. The groups were statistically identical with regard to gender (p = 0.92) and academic ability (p = 0.54) as indicated by one-way ANOVA.

### Student dynamics

In the standard tutorial environment, the students began to participate slowly but engaged with the lecturer more often as it progressed. Students would often begin by writing, but this would diminish as the tutorial progressed. By the time the second case in the standard tutorial had begun, the class would be participating at its peak. Group responses to questions and small to medium scale student-student interactions were common. By the beginning of the third case, small groups were comfortable discussing issues, but the group itself seemed to tire and interactions with the lecturer lessened.

The introduction of the EVS into the second tutorial occurred after a 10-minute break. Students came back refreshed and enthusiastic about using the teaching aid. The interaction with the lecturer ceased at this point and did not return. Students appeared to devote most of their attention to the screen and the EVS controller. Small groups, which had formed in the first tutorial continued to exist and new ones began as students discussed which option was most appropriate to manage the case presented on-screen. This behaviour was consistent throughout the entire second tutorial, although activity did decrease near the end of the last case as students began to tire. Note taking activities throughout this second tutorial were less than in the first.

The dynamics of student interactions during the tutorials with and without the electronic voting system is shown in Table 2. The introduction of an electronic voting system did not affect the length of teaching time required to present material of equivalent length. It did not affect the level of student-student interactions, but did lead to a reduction in the number of students note-taking and the time spent writing. The introduction of the EVS dramatically reduced the number of student-lecturer interactions.

**Table 2:** Summary of student tutorial dynamics, shown as medians

	No EVS	EVS	
Time Spent per Case (minutes)	15	15	ns
Students Participating/case	12	10	p = 0.02
Total Writing/case	10	2	p = 0.006
No. Different Students Writing/case	5	2	p = 0.006
Level 1 Interactions/case	4	1.5	p = 0.003
Level 2 Interactions/case	7.5	1	p < 0.0001
Level 3 Interactions/case	1	0	p < 0.0001
Student-Student Interactions/case	3	5	ns

### Test evaluations

The gender and educational status had no confounding effect and were therefore excluded from the mixed model analysis.

#### Multiple-choice questions

There was a significant 3-way interaction between time, EVS usage and order (p < 0.001) however it was not possible to find any effect consistent with the use of the EVS. The adjusted mean MCQ test scores for both tutorials are shown in Table 3. There was a significant improvement in scores both immediately after and six weeks after the tutorial, irrespective of whether the tutorial included the EVS or not for both tutorial topics. At pre-test for the topic of the acute abdomen, the students who had EVS for this topic had significantly different pre-test scores from those students who did not. This result is likely to be a type II error, as the students had not used the EVS at the time of the pre-test. It is possible that the discrepancy may be due to the reliability of the series of multiple-choice questions used (pre, post and 6 week), which ranged between 0.40 and 0.53. There was a significant difference in Post-Test scores between EVS and no EVS for the gastrointestinal subject, but in light of the unexplained discrepancy at the time of pre-testing, the authors cannot conclude that this represents a real effect. All other comparisons between groups yielded no significant differences regardless of order, nor was there any significant difference in retention (Immediate Post-Test scores subtracted from six Week Post-Test score) between students who had tutorials with and without EVS for both topics.

**Table 3:** Number of correct MCQs from 11 questions, shown as adjusted mean (standard error)

Subject: Acute Abdominal Pain				
Tutorial	Pre-Test	Immediate Post-Test	6 week Post-Test	Retention

No EVS	3.88 (0.24)	5.74 (0.23)	6.22 (0.27)	0.48 (0.28)
With EVS	3.91 (0.23)	5.59 (0.21)	5.89 (0.22)	0.30 (0.25)
difference	ns	ns	ns	ns

Subject: Gastrointestinal Haemorrhage				

Tutorial	Pre-Test	Immediate Post-Test	6 week Post-Test	Retention

No EVS	4.79 (0.23)	7.60 (0.21)	6.51 (0.25)	-1.09 (0.27)**
With EVS	4.20 (0.25)	6.94 (0.22)	6.20 (0.23)	-0.74 (0.26)*
difference	ns	p= 0.03	ns	ns

**p < 0.0001, pairwise comparison for difference over time (t-test)

*p < 0.05, pairwise comparison for difference over time (t-test)

#### Multi-stage questions

The interaction between time, EVS usage and order for the problem-solving questions approached significance (p = 0.07). The adjusted mean MSQ test scores for both tutorials are shown in Table 4. As with the multiple-choice questions, there was a significant improvement in scores both immediately after and six weeks after the tutorial, irrespective of whether the tutorial included the EVS or not for both tutorial topics. There was a significant difference between EVS and no EVS for the post-test scores for gastrointestinal haemorrhage and in the 6-week post-test scores for the acute abdomen. All other comparisons between groups yielded no significant differences regardless of order. There were small but significant benefits observed in retention between students who had tutorials with EVS compared with those who had a tutorial alone. This was true for both tutorial topics.

**Table 4:** Scores for multi-stage questions, shown as adjusted mean (standard error). Maximum score was 12

Subject: Acute Abdominal Pain				
Tutorial	Pre-Test	Immediate Post-Test	6 week Post-Test	Retention

No EVS	3.60 (0.25)	6.24 (0.17)	5.96 (0.20)	-0.28 (0.23)
With EVS	3.97 (0.26)	6.20 (0.20)	6.72 (0.22)	0.52 (0.26)*
difference	ns	ns	p = 0.01	p = 0.01

Subject: Gastrointestinal Haemorrhage				

Tutorial	Pre-Test	Immediate Post-Test	6 week Post-Test	Retention

No EVS	3.88 (0.24)	6.79 (0.15)	5.92 (0.19)	-0.87 (0.22)**
With EVS	3.90 (0.28)	6.22 (0.21)	6.20 (0.23)	-0.02 (0.27)
difference	ns	p= 0.03	ns	p = 0.02

**p < 0.0001, pairwise comparison for difference over time (t-test)

*p < 0.05, pairwise comparison for difference over time (t-test)

### Student opinion

Most students found that the standard tutorial without the EVS was reasonably relaxed, found it stimulating, could easily interact and participate. Only a minority felt inhibited and did not want to join discussions. The large majority considered the tutorial well organised and provided useful and easily understandable information.

The internal consistency of the scale used to measure the students' attitudes towards the EVS was determined using Cronbach's Alpha, which yielded a value of 0.84 (n = 99). The average result from summing the items in the questionnaire was 24 ± 1, where the range of values was from 5 to 30 (30 indicates the maximum positive response to the use of the EVS). The majority of the students felt that addition of the electronic voting system to the tutorial increased the enjoyment and encouraged participation – more so than with the tutorial alone. However one quarter of the tutorial group felt that the EVS made no difference to the tutorial and did not influence their ability to contribute.

## Discussion

This study evaluated the place of an electronic voting system in the structured learning process of an undergraduate medical curriculum. Results showed that when used with students at the beginning of their clinical experience, this teaching and learning aid fostered enthusiasm for the tutorial and encouraged group participation. Quantifiable benefits were limited to a small improvement in retention of understanding of the topics tested.

Active participation in a tutorial has a positive bearing on the educational process. In trying to increase audience participation there are a number of problems to be overcome. These include class size, the cultural backgrounds of the participants and the ability and enthusiasm of the tutor to engage the audience. Whether or not some of these problems can be overcome by means of various teaching aids is debatable.

Different types of teaching aids have been used in the classroom, including audio-visual aids such as slides, movies, PowerPoint presentations, and computers [9-11]. Paper-based tasks such as worksheets and partially completed notes for students to complete during a teaching session have also been used [12]. Studies of the effect of these interventions consistently indicate the lack of effectiveness of a standard didactic presentation and show student preference for some other form of teaching. Many of these interventions are time-consuming and costly to use and it can be difficult to justify their use if they are not shown to have some form of positive educational benefit.

A previous study by the authors used Medici as a teaching aid for a tutorial (unpublished). This study showed that the addition of computer based material into the tutorial made no difference to long-term understanding of material covered in the program. It also showed inclusion of computer-based material increased the time students spent writing within the tutorial. Students perceived the teaching aid to be enjoyable, but many felt it to be too reliant on the ability of the tutor to make it worthwhile. Presentation of material in this form has not been shown to have any negative effect on performance or learning [13].

Electronic voting systems are frequently encountered at meetings and used to measure audience opinion. Whilst it is obviously a more accurate method of counting responses than relying on a show of hands, questions remain on the effectiveness of such systems in engaging the audience and increasing participation. These voting systems are used in student teaching, but little formal evaluation of the process has been undertaken [10,14,15]. The theoretical advantages of an electronic voting system include increased audience participation by allowing anonymous decision-making and by providing immediate feedback to both student and tutor allowing misconceptions to be addressed at the moment they occur. The participants can then see the collective response and as a result may feel less inhibited when contributing to any forthcoming discussion. This could be a two-way process, making contact easier for more diffident tutors. In principle, this may make it easier for a tutor to develop reciprocity and cooperation among students, use active learning techniques, provide prompt feedback and communicate high expectations. These are four of the seven principles of good practice in undergraduate education [16,17] and as such, should be among the goals any tutor sets for a tutorial.

Use of instant feedback devices in tutorials and large class settings in a tertiary environment has been reported in a variety of settings dating back to the late 1960s [10]. The most basic of these is a rudimentary discussion of the answers to multiple-choice questions [18]. A more complex arrangement obtaining more complete feedback from students relies on students holding up coloured pieces of paper to answer true false questions [19] or standard 5 stem MCQs [20]. Electronic response systems have been used in nursing and obstetrics [8,14]. Tutors have reported increased participation from students in these studies and student reaction to these interventions has been consistently positive. Where measures of student performance have been made, the results tend towards a positive effect.

While the students in the present study felt that the EVS fostered participation, the observers noted that the participation changed from student-tutor interaction, to student-student interaction, with relative isolation of the tutor. Although the students reported that the system encouraged them to participate, it reduced direct interaction with the tutor. We have shown that the tutor's role had become secondary to the technology itself and that the type of participation the students had was altered to be more inclusive of fellow students and less inclusive of the tutor. This change in interaction provides advantages for both student and tutor, especially if either party struggles to interact with the other. It is quite possible that the addition of an electronic voting system to a tutorial with a more didactic and passive tutor may have shown objective benefit in short and long term knowledge and understanding of the tutorial subjects by the students. Conversely, this lack of tutor interaction might be regarded as a negative factor for those tutors who enjoy interacting with classes.

It could be that any perceived benefit from using the EVS may be a one-off effect, due to the novelty of the device. This study did not address this issue, but long-term evaluation of a non-electronic true/false feedback device indicates a growing acceptance of using that type of response method and an increase in the perceived benefit of the use of such a system [19]. Thus it is likely that the use of an EVS may provide longer-term benefits as students become more familiar with the use of the intervention.

The observed reduction in note taking caused by the use of the EVS may have both positive and negative aspects. If notes form a pivotal part of the learning process, then the reduced time spent writing was a disadvantage. If less time taking notes meant more concentration on the tutorial and more participation, this could be seen as advantageous. In this study, where there was no requirement for the students to study for any of the assessments, the role of notes being required for revision did not arise. If the EVS is used in tutorials included in formal assessment, it may well be desirable for notes to be provided to students to allow for later revision. The authors have noted previously (unpublished) that the presence of computer aided learning tools in a tutorial has been reported to increase note taking, so the introduction of the EVS may be acting as a balancing effect.

The results of the test measurements in this study demonstrated that the addition of the EVS to the tutorial made no difference to the short term knowledge and understanding imparted but that there was a small increase in long-term retention of problem-solving skills. The increase in long-term understanding is of potential importance, as this is an indicator of stimulus of deeper learning. It has been reported that students involved in problem based learning exercises retain long term understanding better than those who participate in traditional teaching environments [21]. A similar pattern has been observed in this study, where in many respects both the EVS and the case based computer material turned a conventional tutorial into a problem based discussion group.

It has been reported that student attention dramatically decreases after 15 minutes in a lecture environment [22]. One might expect any method, which provides regular stimulus in a class will be of benefit in terms of increasing the attention span of students. A tutorial is intended to provide a continuing intellectual challenge and it may be that the effects of additional teaching aids are lessened due to the nature of the teaching environment. Greater objective benefit may be derived from teaching aids when the class size is larger, or when the tutor is less able to interact with the students. This study was conducted in small group setting because it was planned to study one complete academic year. These students never met as a large class and therefore the only practical way of running the study was when the students were undertaking their six week surgical attachment and could be studied then in a block – albeit a relatively small one. In addition, there was only a limited amount of EVS equipment available – sufficient for 30 students. These are common types of constraints for any form of educational research. In order to test the usefulness of any educational intervention in a real learning situation, compromises are often required.

This study had a number of strengths. First, we were able to enrol the students from a single year of the clinical course and thus ensured an appropriate spectrum of academic ability from a single cohort of students. Second, the study was able to determine clear end-points and apply both quantitative and qualitative measurement in a 'real' educational environment. A weak point – which was recognised when the study was designed and which was unavoidable – was that it would have to run over an entire academic year over six different groups of students and therefore required smaller groups than would be desirable in an ideal situation. The authors feel this may have reduced the impact of the introduction of the EVS. The risk of different groups of students starting with different baselines, depending on how far along the course they had progressed when they did the study was tested and found not to have any confounding effect on the results. Time constraints imposed on the study by the medical curriculum meant that the number of questions, which could be imposed in the testing phase were not quite optimal.

## Conclusion

Teaching aids such as computers and electronic voting systems can be expensive to purchase and maintain. Apart from the initial hardware cost, software must either be bought or constructed and appropriate content developed. If there are no tangible benefits from systems such as the one used in this study, funding might be better used on staff development. Despite this caveat, the future of immediate feedback response systems appears bright. This study has shown that electronic voting systems can provide a stimulating learning environment for students and in a small group tutorial may improve educational outcomes. The wired systems such as the one used in this study are rapidly being replaced by wireless equipment, which should be cheaper, easier to use and more versatile. The growing popularity of Personal Digital Assistants (PDAs) and the expanding capabilities of mobile phones combined with the relative ease of using existing web-based technologies mean that the need for a dedicated response system is waning. The future for this type of system is clearly personal, mobile and adaptable.

## Competing interests

One of the authors (DM) has developed the electronic voting system used in this study. One version of this product (not the version used in this study) is available for hire. At no stage did the author seek to influence the outcome of the study nor did he have any part in the analysis of results.

## Authors' contributions

Funding: EP, PGD, DM

Study Design EP, PGD, DM

Study management and implementation: PGD, NDeY, EP, DM

Initial analysis of data: EP, NDeY

First draft of manuscript: EP, NDeY, PGD

Intellectual input into later drafts and final manuscript: EP, DM, PGD, NDeY

## Contributors

Statistical analysis was completed by Mr. Justin Lokhorst, Dept. of Public Health, University of Adelaide

## Pre-publication history

The pre-publication history for this paper can be accessed here:


